# Becoming the Denigrated Other: Group Relations Perspectives on Initial Reactions to a Bipolar Disorder Diagnosis

**DOI:** 10.3389/fpsyg.2012.00347

**Published:** 2012-09-19

**Authors:** Susan G. Goldberg

**Affiliations:** ^1^Department of Psychology, Duquesne University, PittsburghPA, USA; ^2^Fielding Graduate UniversitySanta Barbara, CA, USA

**Keywords:** bipolar disorder, mental illness, group relations, psychoanalytic, psychodynamic, Bion, Foucault, stigma

## Abstract

The initial reactions to a bipolar disorder diagnosis of research participants in a small, qualitative study consisted of astonishment, dread of being “mad,” and extremely negative associations. All had prior mental health diagnoses, including episodes of severe depression (all but one) and alcoholism (one). All participants reported mental health histories prediagnosis and most had spent years contending with mental health labels, medications, symptoms, and hospitalizations. In addition, most participants were highly educated health professionals, quite familiar with the behaviors that the medical system considered to comprise bipolar disorder. Their negative associations to the initial bipolar disorder diagnosis, therefore, appeared inconsistent with their mental health histories and professional knowledge. This article contextualizes these initial reactions of shock and distress and proposes interpretations of these findings from societal and psychodynamic group relations perspectives. The participants’ initial negative reactions are conceptualized as involving the terror of being transported from the group of “normal” people into the group of “mad” or “crazy” people, i.e., people with mental illnesses, who may constitute a societal “denigrated other.”

## Introduction

… And Something’s Odd— Within—That person that I was—And this One— do not feel the same—Could it be Madness?— This?

(Dickinson, 1862/1960)

Imagine that you are professionally successful. You have faced some psychological challenges, including episodes of severe depression, but you generally perceive yourself as psychologically normal. Then imagine that, as an adult, you are diagnosed with bipolar disorder. How do you make sense of this new label? How do you come to terms with feeling that you have moved into the category of people with mental illness, a group perhaps still considered to be a “denigrated other”? Research participants in a small qualitative study grappled with these questions when they were first diagnosed with bipolar disorder in adulthood.

### Societal group processes

Interpretations will be proposed to make sense of the negative and panicked initial reactions of research participants to a bipolar disorder diagnosis. The interpretations are based on psychodynamic and sociological group relations perspectives. In order to contextualize the interpretations, this literature review discusses theoretical ideas and research findings from both the sociological and psychodynamic fields.

#### Locating deviancy

Sociological labeling theory addresses how societal processes create the distinctions that determine deviance, claiming that society imposes upon select individual’s behaviors that are deemed abnormal, and social institutions then ensure that those designated deviants remain in their assigned roles (Scheff, 1999). These demarcations are strongly enforced and perpetuated: “One sees reflected in categorical devaluation an apparent urge to differentiate as much as possible between “them” and “us” (Schur, 1984, p. 28).

People labeled as “other” have limited options in response, generally restricted to accepting or rejecting the categorization. Labeling theory further posits that people are rewarded for remaining in the deviant role and punished if they attempt to return to mainstream roles. In the deviant role, the person holds a certain status associated with being sick or ill, and when individuals accept the role, they are considered to be “engulfed” into it. Goffman (1961) described the dehumanizing process people encounter when entering the role of mental patient or institutionalized person. They become indoctrinated into the role as their property, bodies, and sense of self are removed incrementally, until eventually they are demoralized enough to comply with the rules of the institution and adopt the negative label assigned to them.

When Goffman (1961) was writing in the 1960s, most institutionalized mental patients did not receive treatment by choice. Psychiatrists had the power to decide what rights and property mental patients would have; psychiatrists had the power to label patients and designate the category of their pathology. Goffman argued that an institutionalized mental patient had to capitulate to the newly created social designation in order to survive in the institution.

Whether people with mental illness labels are institutionalized, as in the 1960s, or remain in the community, as is the case today, sociological theorists contend that society, the media, and individuals continually enforce the distinctions and remind the mental patient to stay in role.

#### Stigma

Labeling theory is premised upon society having negative views about those who are deemed to be “other,” a process referred to as stigma. There is extensive research on the stigma of mental illness. Even today, research has found that Americans continue to hold negative, fearful, or avoidant attitudes toward people with mental health diagnoses, particularly schizophrenia (e.g., Angermeyer and Matschinger, 2003). Corrigan and his colleagues have continually found that mental illness stigma continues unabated (Corrigan, 2005; Corrigan and Watson, 2005; Schomerus et al., 2012). Link and Phelan (2001, 2006) have focused their research on public views of people with mental illness, finding even recently that stigmatizing attitudes persist. Wahl et al. (2002), Wahl (2003), Wahl and Aroesty-Cohen (2010) have studied media portrayals of people with mental illness, concluding that the media perpetuates negative attitudes toward mental illness. These negative views are exacerbated, of course, after every mass shooting by someone viewed as having a mental illness.

Although the stigma of mental illness remains powerful, researchers have found that levels of stigma are starting to vary among different mental health diagnoses. Recent research suggests that the stigma of depression has diminished in recent years but negative attitudes toward mania (often present in bipolar disorder) and schizophrenia have not (Angermeyer and Matschinger, 2003; Wolkenstein and Meyer, 2008). Whether and to what extent the bipolar disorder stigma may have receded has not yet been determined.

#### Reactions of people receiving the label

What the societal research does not address is how people labeled with mental health diagnoses engage with the societal processes that stigmatize, denigrate, and label people as other. Goffman (1961) recognized this concern in proposing that the term “stigma” should have two facets: the societal prejudice toward deviant groups; and the “mark” or personal experience of the labeled person. Following Goffman, there is a significant body of research on how people with mental health diagnoses react to societal pressures and perceived experiences of stigma.

Lally (1989) applied labeling and role theories to the experience of mental patients as they are indoctrinated into the role of mentally ill person. Lally found that the process of role engulfment is not passive, as contemplated by the labeling theorists, but quite active. Lally proposed that in each of several phases of becoming inculcated into the new role, mental health patients were trying to maintain a positive sense of self that contrasted with the negative views of mental illness that surrounded and sought to engulf them.

Other research has found that people who have been labeled with a mental illness regularly experience negative and demeaning interactions with other people, both at work and otherwise. They may fear negative reactions to such an extent that they choose not to disclose that they have a mental health diagnosis (Wahl, 1999; Goldberg et al., 2005; Link and Phelan, 2006).

The stigma, labeling, and role research examines attitudes, views, perceptions, and other relatively conscious activities. Psychodynamic approaches are therefore useful to investigate that which is unspoken, unknown, and unconscious.

#### Psychodynamic conceptualizations of group process

The psychoanalytic group relations theories advanced by Wilfred Bion, expanding upon ideas developed by Melanie Klein, focus on group unconscious behavior (Bion, 1959). In Kleinian/Bion theory, when the infant projects difficult material into the mother and the mother can “contain” these feelings, the infant can then experience and metabolize the feelings safely. However, if the mother cannot contain them, the child feels intense terror and unraveling, ultimately projecting the powerful negative feelings outside of itself and locating them in another. This projective identification process is repeated, in a kind of parallel process, in group behavior.

Working with Bion’s ideas, Horowitz (1985) proposed several group processes that utilize the notion of projective identification. Two are important here: “role suction,” which is using a member as a spokesperson, and scapegoating (Horowitz, 1985, p. 29). Role suction is the process by which a group places one of its members in a specific role and then deposits projections into that person. In scapegoating, one member of the group is used as a spokesperson, particularly for socially unacceptable causes. That member comes to claim all of the negative aspects of that denigrated position, allowing the other members to adopt an attitude of superiority and benevolence toward the scapegoat. In the context of projective identification, scapegoating involves the displacement into the scapegoated person of all the group’s “unwanted affects” and “desired but threatening impulses” (Horowitz, 1985, p. 30). These processes then manifest in groups in society; members of these groups ultimately become filled with these negative societal projections (Main, 1985).

Another approach to consider how society forms “us” and “other” groups involves noting that any person, except mother, is an “other” to the developing infant (Erlich, 1997). The stranger then represents “the prototype of the internal psychic enemy that becomes a social reality,” and all strangers or enemies hold a “boundary position” (Erlich, 1997, p. 133). In fact, “others” who are most like us are often our most hated enemies (Volkan, 1986). By maintaining ties to the “enemy” through projective identification, we maintain a peculiar ongoing relationship with that particular enemy and may even become obsessed with its members. Volkan’s exploration of how groups maintain enemy groups, often coming from our closest neighbors, has a psychoanalytic lineage, starting with Freud:

Each individual is separated from others by a “taboo of personal isolation,” … and it is precisely the minor differences in people who are otherwise alike that form the basis of feelings of strangeness and hostility between them (Freud, 1917/1961, p. 199).

From a psychodynamic perspective, the closer the enemy is to us, the more we need to create rituals, symbols, boundaries, and cultural artifacts to separate ourselves from this group, perhaps different from us in only minor ways, even as we remain connected to them through the process of projective identification.

Based on these societal processes, groups in the dominant position create and maintain groups of denigrated others (Green and Skolnick, 2002). Those placed in an outgroup are containers of our displaced and projected negative affects. Expanding upon that idea, Altman (1995) analogizes the psyche to neighborhoods: there are idealized good and denigrated bad neighborhoods. Bad people are in the bad neighborhoods; people from “good” neighborhoods avoid them for fear of what happens there. Through group process, “the public sector becomes the repository, on the social level, for the ‘not-me,’ for the disowned, the different, the degraded, the incomprehensible” (Altman, 1995, p. 127). The questions here are whether people with mental health diagnoses remain in a denigrated other category in American psychic life and, if so, to determine which affects have been placed onto them by the majority.

#### The location of madness

Foucault (1954/1976) proposed an answer to these questions. Expanding on the psychoanalytic perspectives, he noted that societies continually locate “mad” people away from “normal” people. Foucault (1965/1988) noted how in Western society, this need for separation was once manifested in physical displacement. He argued that during the Renaissance mad people were separated from the rest of the populace. Foucault used the example of the “Ship of Fools,” a likely mythical ship for mad people who were removed from cities and towns. The ship was believed to travel up and down rivers; the mad passengers could not leave, like prisoners with a life sentence. For Foucault, the metaphoric and symbolic power of a Ship of Fools represents society’s desire to place mad people at the threshold between the known and the unknown, so they become located both outside (outside of towns) and inside (in rivers that are inside of land) the known. Mad people could then contain for the dominant group all of our fears of the unknown.

Foucault (1954/1976) provided one explanation for how Western society deals with those people placed outside the “normal” threshold:

… our society does not wish to recognize itself in the ill individual whom it rejects or locks up …. The analyses of our psychologists and sociologists, which turn the patient into a deviant and which seek the origin of the morbid in the abnormal, are, therefore, above all a projection of cultural themes (Foucault, 1954/1976, p. 63).

The American “cultural themes” of madness that are projected to the outside world involve out of control and crazy behavior. Such cultural notions that contrast psychological normalcy with madness might seem outdated. The question remains, however, whether these ideas continue to be ingrained in Americans’ underground cultural and psychic lives. Exploring these questions through interviews of people diagnosed with bipolar disorder in adulthood provides a glimpse into how these unconscious processes may play out today.

#### Bipolar disorder

Psychological literature today is replete with articles about bipolar disorder, including its etiology, treatment, and the biochemical imbalances requiring correction (e.g., Kurtz and Gerraty, 2009; Lönnqvist et al., 2009; Swartz et al., 2012). Yet discussion on how stigma and other societal processes affect people labeled with bipolar disorder has been minimal (Proudfoot et al., 2009).

## Materials and Methods

### Research question

The overall study investigated the construct of bipolar disorder in the United States today, exploring how it represents a particular set of feelings, experiences, and behaviors; how society explains the construct; and how those explanations may have changed over time. I examined the questions through the experience of several individuals diagnosed in adulthood with bipolar disorder. This paper addresses one area of the findings, i.e., an analysis from group relations perspectives of the intense and negative initial reactions participants had upon receiving the bipolar diagnosis. I have also proposed an analysis of other areas of the findings, through the lens of social constructionism (Goldberg, under review). There I explored the vagueness of society’s view of the threshold between normal and hypomanic behavior, how that blurry threshold impacts upon people diagnosed with bipolar disorder, and how today’s constructions differentiate between depression and bipolar disorder.

### Informed consent

The study was approved by the Fielding Graduate University Institutional Review Board. Informed consent was obtained from all participants and is archived by the author. In order to protect the confidentiality of participants, their names, along with some identifying information, have been changed.

### Recruitment

People diagnosed in adulthood with bipolar disorder were the target population, particularly people who had thought deeply about the meaning of the diagnosis in their lives. The study was my dissertation research. I distributed flyers to classmates at Fielding Graduate University, who referred seven people, six of whom became participants. The Fielding doctoral students knew the participants as therapy clients (three); an acquaintance (one); a former clinical supervisor (one); and a professional colleague (one). Throughout the research, I remained sensitive to how this recruitment process resulted in a rather homogeneous group of participants, by virtue of both my particular recruitment request and the participants’ links to my Fielding colleagues.

### Interview process

The interviews, which were approximately 2 h long, were open-ended to allow participants to respond to the research question in a stream-of-consciousness style. The first two interviews, which were pilot interviews, were divided into two shorter sessions held roughly 1 week apart. All interviews except one were conducted in person; one interview took place by telephone. This participant used a pseudonym due to her concerns of adverse professional repercussions if her identity were known.

At the conclusion of each interview, participants provided the demographic information listed in Table 1. None of the participants had a clear recollection of all of the information requested. For example, some could not remember the specific year of their initial diagnosis of bipolar disorder or the names and dates of prior mental health diagnoses. Thus, Table 1 should be understood as reflecting participants’ best recollections.

**Table 1:** **Basic demographic information about participants**.

Name	Age when interviewed	Date of bipolar disorder diagnosis	Previous diagnoses	Age when received previous diagnosis (if recalled)	Ethnic identity	Occupation
Darlene	48	40	Anxiety and Depression, Borderline Personality Disorder, Co-dependency	Anxiety and depression, about 34	European American identity (ethnicity identified as Caucasian and half-Arabic)	Nurse-practitioner (not currently working, mostly due to bipolar disorder diagnosis)
Jodi	45	40	Depression	38	European American	Physician – internist
Kevin	39	30 or 31	Depression	Could not recall age	European American	Clinical psychologist
Natalie	46	44	Alcoholism	Since childhood; no formal diagnosis in adulthood	European American (ethnicity identified as German, Scotch-Irish, and 1/64 African American)	Government worker (unemployed at time of interview)
Rose	55	54	Depression	44 or 45	European American (ethnicity identified as Irish–Welsh)	Manager and administrator, government and private sector (not working at time of interview, mostly due to bipolar disorder diagnosis)
Sarah	40	25 or 26	Depression	15	European American	Clinical psychologist

### Data analysis

Interview recordings were transcribed and analyzed using the epistemological approach of hermeneutics, as articulated by Gadamer (1976/1992) and Taylor (1989), who claim that interpretation occurs in a cultural context. From this perspective, it is not possible for the interpreter to step away from her or his perceptions and biases to develop an objective account of the facts, especially in a social science investigation that seeks to understand meaning-making. In fact, our individual knowledge and reactions may be necessary to understand and interpret a phenomenon. This approach conceptualizes multiple interpretations of a phenomenon, with none being more correct than another (Janesick, 2000; Schwandt, 2000).

According to Ricoeur (1970), the hermeneutic approach can be considered from two perspectives, the “hermeneutics of faith” and the “hermeneutics of suspicion” (Josselson, 2004). The hermeneutics of faith, or of restoration, involves representing the voices of the participants as richly as possible; from this perspective, I sought to bring to the reader the subjectivity of those labeled with a mental disorder. The other approach is to analyze and interpret that of which participants may be unaware. Ricoeur called this approach the hermeneutics of suspicion or demystification (Josselson, 2004). My analysis moved between these two positions.

I also grounded my approach in social constructionism, which posits a more ephemeral truth. These two approaches can be contrasted: “philosophical hermeneutics trusts in the potential of language (conversation, dialog) to disclose meaning and truth,” while in social constructionism, “there is no truth to the matter of interpretation” (Schwandt, 2000, p. 198), because all meaning is created solely through relationship (Gergen, 1994). In other words, to perform a social constructionist analysis, societal and cultural beliefs, norms, ideologies, and politics are requisite for interpreting experience.

### Demographics

The six participants were diagnosed with bipolar disorder as adults, some within a year of being interviewed. All participants were educated and professionally successful: two were clinical psychologists, one was a physician, one was a nurse-practitioner, and two held responsible government or business positions. At the time of the interviews, three participants (the psychologists and the physician) were working in their professions, while the three others were not working, due to bipolar disorder symptoms or bipolar medication reactions.

Participants were five women and one man. Four were married; one married after the interview was conducted; and one woman is divorced and now dating another woman. At the time of the interviews, four of the participants were in their 40s, one in her 50s (Rose), and one in his late 30s (Kevin). All participants identified as European American, with two reporting additional ethnic identities (one was half-Arabic; another reported an African American ancestor).

The age of diagnosis ranged from 25 or 26 to 54. All but one of the participants had prior psychiatric diagnoses; five of the six had prior depression diagnoses. The sixth, Natalie, was in treatment for alcoholism when diagnosed with bipolar disorder. At the time of the interviews, all were taking one or more psychiatric medications for bipolar disorder, including Depakote, Resperidol, and Effexor. Prior to the bipolar diagnosis, one (Darlene) had been hospitalized three times for mental health issues, one (Rose) had a brief hospitalization after a possible suicide attempt, and two others had been hospitalized following suicide attempts (Jodi and Sarah).

This is a purposive, not a representative, sample. There is little demographic diversity among the participants as all identify as European Americans, are middle-class, and are professionally successful. There is also limited gender and sexual orientation diversity, as five of the six are women and five of the six are heterosexual (one divorced woman now identifies as a lesbian). It is also not a representative sample in terms of people with bipolar disorder. In the language of psychiatric treatment, most of the participants would probably be considered reasonably “high functioning.” This demographic information is laid out in Table 1.

## Results

### Being labeled

Being assigned the label of bipolar disorder was an experience of crossing over the threshold from normal to abnormal and was a devastating moment for participants. As in the Emily Dickinson poem above, they feared that the bipolar label indicated they had suddenly become mad. The participants moved through a series of reactions to the initial diagnosis, yet the first reaction, across the board, involved shock and despair.

#### The initial shock

The participants used strong words to describe their initial reactions. Jodi, an internist, reported being flabbergasted, even though she had been having challenging affective experiences prior to the diagnosis. Her first psychiatrist hinted that Jodi might have bipolar disorder but did not give a definitive diagnosis:

She said, “Did anybody ever tell you that you might have bipolar disorder?” And I—, my jaw just, like, DROPPED, and I was like, “NO.” I mean it never even dawned on me that—. WHY it never dawned on me, I didn’t know.

A few months later, Jodi had an episode at work when she was crying uncontrollably. The next day, she went to a colleague of that first psychiatrist. She described the shock she felt when the second psychiatrist definitively diagnosed her as having bipolar disorder.

That visit was one of the most painful things that I have ever experienced. … The physician that diagnosed me that day was very uncaring. He really had no clue what he was saying to me and what kind of impact this was having on me. Absolutely none. And basically sat there with me and said, “This is what you have. You’re going to need to be on medication for the rest of your life. … Here’s a book to read. Here’s—, go to this support group. And off you go.” Trot trot.

The sense of being dismissed is captured in Jodi’s phrase, “And off you go. Trot trot.” Yet the most devastating aspect for Jodi seems to be the fear that she was no longer normal:

You have to understand what a tremendous blow to my ego it was to be diagnosed with bipolar disorder…. You cannot live the rest of your life like other people. You … are different than other people. … The bottom line is, you’re not NORMAL [laughs]. … It was really hard. It’s no wonder it’s taken me like five years to even to get to a space where I can go, “OK, I’m doing all right now.” … It’s taken all of this time, really, to kinda get my feet underneath me and just to be able to … even talk about it in a way that I understand.

Jodi felt that it took 5 years to recuperate from receiving that heartbreaking news. Her shock may be difficult to understand if we remember that Jodi is a physician who presumably would have been aware of the symptoms the medical system deems evidence of bipolar disorder. Moreover, her father had bipolar disorder. Yet she is not alone in her surprise.

The first suggestion that Sarah, one of the clinical psychologists, might face bipolar disorder was from her mother’s psychiatrist, after he diagnosed her mother with bipolar disorder. Sarah said she “fought it tooth and nail” even though “his reasoning was very sound.” He felt that, because Sarah’s mother and grandmother were diagnosed with bipolar disorder, Sarah may well have it too. Sarah’s reaction to the long-distance diagnosis by her mother’s psychiatrist both irritated and frightened her. “It pissed me off. It probably scared me too, but I don’t think I knew that it scared me.” Kevin, the other clinical psychologist interviewed, also had a strong reaction to the diagnosis. He indicated that he was “devastated” when he received the diagnosis, which was about 7 or 8 years before his interview.

#### A “crazy” diagnosis

The participants’ initial response was that bipolar disorder is a crazy diagnosis, unlike depression, and this meant that they *were* crazy. The initial reaction by Rose, who had worked as a high-level administrator, was that it meant she was–

nuts. Manic. A manic depressive is somebody who’s institutionalized. A manic depressive is completely out of control. Somebody that has to be locked up. Or is doped up so bad that they really can’t even function anyway, except they can’t hurt themselves.

Rose noted that in her culture of origin, one would never admit to having a mental health problem. In fact, “even when my family had cancer, we said it in a whisper.” She noted that she still chokes on the word “cancer,” which is not even as negative in her mind as mental illness. “You whisper them, nobody knows. Nobody can find out. It’s something you gotta hide and it means you’re crazy. It means you’re crazy [slower].” For Rose, bipolar disorder means:

I didn’t want to be the lithium-laced wack job who has to be drugged in order to have a—, have a life, because I’m mentally ill. I don’t want to be mentally ill. … I was in an institution for 24hours …. I was with people who ran up and down the hall thinking that bombs were landing on him from Viet Nam. The woman in the bed all night screamed and cried. They took away my shoelaces [laughs]. They took away my watch. … That’s what crazy people are.

Rose’s perceptions of mental illness and bipolar disorder are terrifying. They are associated with people who are psychotic, traumatized, and out of control, associations linked to her one-night stay in a mental institution after a possible suicide attempt.

Natalie, another former high-level government employe, saw a psychiatrist as part of her rehabilitation from alcoholism. She said a psychiatric visit was recommended in order to obtain medication to ease the detoxification from alcohol. Instead, the psychiatrist labeled her as having bipolar disorder. She thought it meant she was crazy:

I didn’t think anything was wrong with me except that I drank. I was a drunk, you know. … So I went to Dr. X. Then in the process of my intake, and two visits later, he tells me, “You know, we really need to treat you for this—, situation. The situation is I believe you’re bipolar” [said in a deep pedantic voice].

Interviewer: So how did it affect—. What did you hear when he first said that?

Nuts [laughs]. It’s what I heard. I heard, “Oh, the doctor’s telling me I’m nuts. That’s nice. I’m going to go back and drink.” But I didn’t.

Notice the colloquial terms the participants are using, like “nuts,” “wacked,” and “crazy.” These words are prominent for their pejorative tone, considering the level of education and articulate expression of all of the participants.

#### Initial rejection

Each participant initially dismissed, denied, or rejected the diagnosis. Rose thought to herself, “No, I’m just depressed” or “I’m just happy.” Rose’s initial reaction to the diagnosis moved through phases of denial, all of which took place while the psychiatrist was speaking. Describing the phases, Rose said she first rejected the diagnosis. Next, she decided that she would be “nice” and simply listen to the doctor. She then told herself that psychiatrists overdiagnose. “And, boy, they just have to stick labels on you.” She then told herself that her psychiatrist was “trying to make too much out of this,” and unnecessarily “going to extremes.” She was thinking, “Prozac’s fine, because it was fine last time.” She then reminded herself to be pleasant to the doctor, because the doctor is “free. How can you argue with free?”

As the doctor described the symptoms, however, Rose could not help but notice that the symptoms did seem to fit her experience. Rose started to feel terror, like “the frog in the boiling water.” Then, much like a guilty sinner, she concluded that she needed to be punished for past misbehavior:

Oh my God! Oh my God! [said slowly] That is me. Because I’m denying it at first. Because your—, my memory … is very selective. Some of the things I remembered here from way, way, way back when I had forgotten I did. So, yeah, OK. I’m fine. I did tha—. Erase that past. Erase that past! I was a bad girl or did something. But no, I’m fine now. I’m fine now [very fast]. So I don’t have to remember that.

Note how Rose refers to selective memory and ends with wanting to forget her “bad” past behaviors, and that one criticism of the psychiatrist was that she was “going to extremes,” one of the very symptoms the psychiatrist was attributing to Rose. This suggests that Rose was unconsciously “taking in” the words and their meanings, even while consciously rejecting them.

Natalie also initially rejected the diagnosis. She did not know much about bipolar disorder and thought of it simply as “yippy skippy.” She recognized that she was a functional alcoholic for many years, but when she was diagnosed with bipolar disorder, she said to herself, “I’m not manic depressive. You know, there’s no way. Cause that’s just NOT-me.” For Natalie, the initial rejection of the diagnosis was easy. Matters became more complicated for her later on.

Over time, the participants progressed through other reactions to the bipolar diagnosis, ultimately finding some relief in the label and eventually coming to a truce with the diagnosis. This paper focuses on their initial reactions in contrast to their prediagnostic understandings, as these reactions seem particularly powerful and compelling.

### Prior negation of bipolar diagnosis

Some of the participants noted that a psychiatrist had previously mentioned the words “bipolar disorder,” but it was mentioned in the context of the psychiatrist stating that the participant did *not* have bipolar disorder. Psychiatrists assured the participants that they did not need to worry. For example, Rose indicated that she “had heard” the word “bipolar” when she was briefly hospitalized 10 years earlier. She was relieved when they only diagnosed her as depressed:

They bantied [*sic*] that word around, as they talked to me, but never actually said that was what was wrong. They went back to the word “depression.” Ten years ago. And put me on Prozac. … My diagnosis was always just depression. So I heard the word manic-depressive and bipolar but let it go because of what I knew society says about manic depressives.

Rose indicated that she was well aware of the negative views of bipolar disorder, and was therefore quite relieved to “let it go” when the psychiatrists assured her she did *not* have that dreaded disease.

Similarly, Darlene was diagnosed as depressed approximately 15 years before being interviewed. As with Rose, the doctor made sure she knew he did not consider her to have bipolar disorder:

I had been put on lithium a few years before that, just as an adjunct of therapy to the Prozac. And I remember that my doctor said, “Now I’m not putting you on lithium because you’re bipolar, because that’s not what I think.” Like—, he was like, “Don’t get worried, I’m not considering you bipolar. I just wanna try this lithium to help the Prozac.”

For Darlene, it was reassuring that the doctor did not consider her to have bipolar disorder, and she has a clearer memory of being told she does not have bipolar disorder than when she was told she does:

I don’t recall the very moment that the words [bipolar disorder] were said to me, because they had been said to me many years before, to say, “No, no, you probably don’t have bipolar.”

The initial mention of the diagnosis and dismissal by their doctors was fuel for the participants’ reactions when they were later diagnosed with bipolar disorder – it suggested that now they *did* need to worry.

### Life prebipolar diagnosis

These initial reactions should be understandable to most readers. We might all imagine the shock and distress that accompanies being labeled with what is considered a serious mental illness. These reactions are inconsistent, however, with the participants’ reports of prediagnostic life experiences. In order to contextualize their reactions, this section describes some of the participants’ early lives and their prediagnostic understanding of their emotional experiences. The participants experienced early childhood traumas, difficult childhoods, and substantial mental health issues prebipolar diagnosis, experiences that participants later viewed as early symptoms of bipolar disorder. The key point here is that, even with these earlier difficulties, the participants never considered that they might belong in the group of people labeled as mentally ill until they actually received the bipolar diagnosis. Rather, they saw their experiences of depression, isolation, suicidality, or other challenges as painful, yet normal, life experiences.

#### Early trauma

All of the participants described difficult childhoods. Three of the six lost a brother during childhood. When Natalie was 4 or 5, her 9-month old baby brother died. He:

suffocated with a cleaners’ bag when he was sleeping. And my Dad blamed my Mom for it. And my Mom blamed my Mom for it. … Nobody was to blame but you know …blame happens. … She didn’t find any need to live after that. … I remember. I saw him, all blue and, um, icky.

Natalie believes that her mother drank herself to death during the next 5 years. Natalie was 10 when her mother died.

When he was a child, Kevin also lost a baby brother. He mentioned this in passing, in an effort to explain why he believes his father criticized him whenever Kevin expressed any exuberance:

We had a car accident when I was 18 months old and my 6-month old brother was killed and both my parents … went through the windshield …before lap/harness belts. And Mom was holding the baby. And so, a—, the world can be a dangerous place, you know. And [my father] really learned that lesson in spades. So whatever lapse of control he had exerted over—, I’m sure that fit with his own dynamic to make him even more afraid and anxious as he then dealt with me.

Kevin continues to wonder if his own hypervigilance about showing any strong emotion stems from the experience of his father’s anxiety when dealing with Kevin’s displays of excitement.

Rose lost a 17-year old brother to cancer when she was 10, one of a long litany of early traumas. These early losses organized much of these participants’ psychological lives.

#### Parental mental health problems

Several of the participants described how the significant mental health issues of a parent affected their early lives. Rose spoke at length about her mother. “She was a … thief and a liar and a cheat. … My mother was a shoplifter.” Rose described how her mother’s moods would fluctuate from the energetic shoplifter to a severely depressed person. “My mother was very, very depressed all the time. If she wasn’t one way, she was another.” Her mother would engage in borrowing, stealing, and spending sprees and at other times, would disappear into her room, leaving Rose to care for the younger children. Rose’s conceptualization of her mother is that she was mentally ill:

There’s just certain things that just stick out in your mind. You know, I mean she was—–, she was all chouchouchou [using hand motion at her ear to indicate mental illness], you know, and I never gave it a name except … mom was wacked^1^.

When Rose was 16, with her mother in a mental institution, Rose became pregnant and married her high school sweetheart. Rose said, “I backed out of my family … because of the alcohol and the craziness.” Rose described most of her siblings as both severe alcoholics and as crazy. Along with the death of her brother, the “wackiness” of Rose’s mother was a central part of her childhood narrative.

Like Rose, Sarah’s childhood was colored by her mother’s mental health problems. At the very start of the interview, Sarah responded to the initial question about receiving *her own* bipolar diagnosis with her *mother’s* history:

Well, let’s see. My mom was depressed when I was growing up. And, you know, was in … psychotherapy for a long, long, long, long time. But then nothing really seemed to help her that much. She just kept being depressed and depressed and depressed. And when I went away to college, she had a manic episode that really had psychotic features, and had to go to the hospital.

Sarah’s foremost memory from childhood involves her mother’s depression. Sarah views herself as having overidentified with her mother but she also felt uncared for, as her mother had limited energy to attend to Sarah’s needs: “I was more like a neglected kind of child.”

Darlene also described difficult parental figures, as she lived with a “crazy” mother and a controlling grandmother.

I had a wacked-out mom. Well, she was pretty much abused when she was a child. … Not a very loving person. … And my grandmother, her mother, lived with us, from the time I was 2. And she was a cold and conniving witch. And I lived with her my whole childhood, ‘til I got out on my own. And she was just a very horrible person. Um, no boundaries. I never had a moment where she didn’t barge in on me or do something. There’s nothing horrible in my background like child abuse or sexual abuse or anything like that. Just had a really unhappy, miserable time.

For this group of participants, navigating a childhood with a disturbed mother significantly impacted their adult lives.

#### Isolation in childhood

Some participants highlighted a sense of isolation in childhood. For example, Sarah recalls early difficulties interacting with other children. “When I was maybe 6, 7, I was taken to play therapy. And I think it was because I was having trouble getting along with … kids in school and I was having temper tantrums.” She remembers being ostracized at school:

I was the kid with the cooties. …The worst kid with the cooties. [laughs]. … Um, you believe you’re the worst kid that there is, and … you just believe it. … And also I wasn’t very popular. I was pretty confused….

Her childhood understanding of her emotional life was linked to this ostracism:

When I attributed anything to anything, it was, “I’m sad because nobody likes me and nobody likes me because I’m ugly and I can’t play sports and I can’t make friends, blah blahblah” [laughs]. … I don’t think I would have believed anybody if they said, “It’s because you are depressed.” I would have said, “No. I am depressed because I have reasons to be” [laughs].

Like Sarah, as a child Jodi was “moody,” “withdrawn,” “shy,” and “I felt very much like … nobody liked me.”

I didn’t have an easy time making friends, at all. I felt very separated from the rest of the children. I just couldn’t seem to easily … fit myself into the social life. … It was like going into a social situation like school and I just wasn’t well liked. And I don’t know why.

These narratives of early loss, managing parental difficulties, and early isolation were offered by participants in response to being asked their experience of receiving the bipolar disorder label. The participants seemed to view these early difficulties as relevant to bipolar disorder, although the connection may not have been easy to articulate.

#### Labeling of moods

Many of the participants described mood fluctuations throughout their lives. All but one of the participants self-labeled with depression, diagnoses later confirmed by psychiatrists. Kevin, for example, reported having “periods of low energy and withdrawal,” which a psychiatrist diagnosed as depression. Rose noted that her moods would fluctuate from high energy to exhaustion. As an adult and a parent, when she locked herself in her room, Rose would be “crying, sleeping. I was an award-winning sleeper.” She would be thinking, “My life was—, zero. My life was nothing. … I used to think the depression was because I had no value …. I just had a feeling of worthlessness.”

Like the other participants, Jodi depicted difficult childhood experiences, describing herself as a “moody child”:

It’s not like this came and reared its ugly head in my 30s. I would have described myself, if you had asked me, as a moody child. Did I know what that meant? Did I know why I had that? No. Absolutely not. I had no clue. But I can remember that I was always super sensitive to criticism and cried very easily. That I was very shy. That I felt depressed and sad a lot of the time.

When Jodi was asked how she made sense of her experience before the label of bipolar disorder, she responded with how others viewed her:

I was aware that people sometimes saw me as a little bit different, different than other people. … I didn’t handle things as well as other people. …I wasn’t as happy as other people. I knew people saw me that way, as tightly wound up and as kind of pessimistic and blue.

Jodi understood her experience by calling it moodiness, anxiety, a mild depression, and being “a little bit different” from other people. Looking back (from the vantage point of bipolar disorder), Jodi is aware that her moods and energy fluctuated. Until she received the bipolar diagnosis, however, Jodi had never viewed these feelings, moods, and behaviors together as a package of related symptoms or experiences. Instead, she labeled each individual experience, such as being depressed, overly sensitive, or unhappy.

Three participants (Jodi, Sarah, and Rose) made suicidal gestures or attempts prior to receiving the bipolar diagnosis. Rose minimized the suicidal gesture she made as a teenager, and could not say if a later event was a suicidal gesture. The second incident occurred about 10 years before the interview, when Rose was having a difficult period that she referred to as “going around the bend.” During this time, “I pulled my car in front of a tractor trailer and smashed up my brand new Jeep Grand Cherokee.” Although she did not indicate that she was suicidal when she drove into the oncoming truck, her doctor hospitalized her in a mental institution and gave her a diagnosis of depression.

Sarah made a suicidal gesture or attempt when she was 15. Although she was aware of suicidality, she also minimized this teenage gesture:

I knew that I wanted to kill myself a lot of the time so, yeah, I think pretty much. [laughs]…. I wound up attempting suicide once. … I was 15…. What I remember, it was a silly gesture, I think. I think I was very angry, more than dead set on killing myself. I took a whole bottle of my mom’s sleeping pills. And she was furious at me. Really, really angry. My mom gets angry when she’s scared. So she took me to the hospital. I wasn’t treated very well. I think it was like a, you know, dumb teenager react—, reaction. They were mad at me. Everybody was mad at me [laughs].

In college, Sarah labeled herself as depressed, yet did not pathologize the depression; she believed that “burning out” was expected for students, like herself, who studied hard; for her, this explanation was sufficient. As an adult prebipolar diagnosis, Sarah generally perceived herself as depressed and temperamental, saying that her husband “always knew of me as a very depressed and sort of tempestuous person.” Those words seemed to capture Sarah’s experience of herself as an adult.

Jodi had a very difficult period in her late 30s, when she was a mother of three young children and in a troubled marriage. She was starting to spend time away from her family, drink heavily, and do odd things, like meeting endless people online. She was diagnosed as “having an episode of depression,” which she attributed to the stress in her life; however, 6 months later, she attempted suicide and was hospitalized overnight. Jodi tried to explain how she was feeling at the time:

There’s no out. There’s just no way to get out of this situation. … I was like, it’s not going to get any better. You’re going to feel this way. You can’t escape this marriage. You’re miserable. You’re gonna have to stay. … You’re stuck. … But you’re miserable. So how you’re gonna deal with that?

Shortly thereafter Jodi made the decision to become sober, a process that started an avalanche of changes in her life, including the diagnosis of bipolar disorder.

Darlene also understood her emotional difficulties as depression.

It’s tiring to talk about. …Of those years. Those horrible years. … Well, it’s exhausting just to be sick all the time. … Every November the depression would just start, you know. Sort of like seasonal affective disorder. I would just get so depressed and would just pray that I would make it through to April when the weather would get a little bit better. ….

Like many of the other participants, Darlene now frames her understanding in psychological and “bipolar” language, like “seasonal affective disorder.” She also recalled experiences that she now labels as manic or hypomanic, which at the time, she thought her “head was nowhere” and “not where it was supposed to be.”

Everything was so hard. And then there would be that crazy, hyper, talkative, mind-racing [sigh]. I would forget to pick the kids up at school because I would be spending money in a jewelry store. And they would have dentist appointments and I wouldn’t even get to the school to pick them up ‘til after the appointment would have been over. My mind was just completely mixed up and I would finally get to their school and they’d be standing, crying. And [sigh], “Where were you?” … [sighs]. My head was nowhere. I mean, it was just not where it was supposed to be. It was exhausting.

When Darlene was in her 30s, she was treated for depression and anxiety and hospitalized three times for mental health problems and suicidal ideation. “I had been hospitalized two times before finding out that I had bipolar disorder. And both times were for serious, severe, major depression.” The years of depression and anxiety were exhausting for Darlene, yet she remembered being very happy when she was not depressed. “I loved life. It was awesome. I was a very outgoing person. I was a very life-of-the-party type of person. Loved it, people. I was all—, we had so much fun.”

Besides depression, anxiety, and joy, Darlene also talked about many episodes throughout her life of intense anger, starting at about age 14. She expressed shame about her tendency to get angry:

When I was 14, that’s when I changed. Because I abused my mother verbally so terribly when I turned 14. From then on, I would just snap. And I would just—. I would use the “f” word all the time. I would hit her. I would push on her. I would scream. I would yell. People walking down the street could hear us. And at that time I thought, she was driving me crazy! It’s not my fault. But when I look back on it, it was NOT normal. It was NOT normal. And, I think that was … the start of it all. And I have done that to my husband for the last 22 years we were married. Off and on, not very often anymore… I think it’s me. I do. It’s terrible. It’s horrible. I’m so ashamed of it. It’s just awful.

Darlene feels shame about her anger but not about the range of other feelings and moods that she later attributed to bipolar disorder.

No one took over the role of parent following the death of Natalie’s mother. Instead, Natalie’s older sisters introduced her to alcohol and drugs, and Natalie drank from age 12 to age 44 when she chose to become sober. She received the diagnosis of bipolar disorder immediately after “detox and rehab” from alcohol. Her primary label for herself during all those years was as an alcoholic. The only participant who did not label her experiences as depression, Natalie did report feeling emotionally “dead” and she “didn’t care about anything” after the deaths of her baby brother and mother, experiences consistent with both grief and depression.

#### No concern about mental health issues for self

What emerges from these narratives of depression, anxiety, and occasional suicidality is that both family members and the medical establishment treated these incidents, prebipolar diagnosis, as “dumb teenager reactions” or as minor affective symptoms. The authorities minimized the behaviors, allowing the participants to view themselves as mentally normal.

For these reasons, the participants never considered themselves as mentally ill, even when they experienced intense mood fluctuations and received labels of depression and alcoholism. Rose, for example, called her mother and many of her siblings “crazy,” “nuts,” “wacked,” and “alcoholic.” Even though she even referred to herself as “wacked” and “crazy,” both in jest and not, she rarely thought seriously about the ramifications of being seen or labeled as “crazy” until she received the diagnosis of bipolar disorder.

## Discussion

The central question of this paper is why this group of people reported feeling such shock and dismay upon receiving the bipolar label. We might expect anyone receiving a diagnosis of a significant mental disorder to be upset and shocked by the diagnosis and to feel overwhelmed by the medical model of mental illness, with its psychiatrists, labels, medications, and hospitals. Yet each of these people had received – and had been comfortable with – one or more prior mental health diagnoses and had been involved in the mental health world. Five had lived for years with medical diagnoses of depression and the sixth with alcoholism. Several had been hospitalized for mental health issues; three had made one or more suicide gestures or attempts; and all had experienced difficult, painful, fluctuating moods. Moreover, four of the six were highly educated professionals in health-related fields: two were clinical psychologists; one was a physician; and one was a nurse-practitioner.

Considering the emotional histories and professional education and training of this group, one would *not* expect that a bipolar diagnosis would feel particularly surprising or upsetting. Instead, one would assume that this group of participants (excluding, perhaps, Rose and Natalie, who did not work in the medical field) were familiar with what the medical system considers symptoms of bipolar disorder and they would have linked with bipolar disorder their mood fluctuations and other experiences that did not fit neatly into a depression diagnosis. Yet none reported considering the possibility, prediagnosis, of bipolar disorder. Although the participants later found the bipolar label to be helpful in organizing aspects of their life experience, both pre- and post-diagnosis, their initial panicked reactions are the present focus.

### Lacking fear of mental illness

Even with formal diagnoses of depression (five participants) and alcoholism (one), the participants did not pathologize their behaviors or have concerns that they might be seen as mentally ill. Their feelings and behaviors generally made sense to them and although their affect or experiences may have been troubling, the participants did not consider them deviant. Rose, Sarah, and Jodi could articulate the logic behind their suicidal gestures or attempts. Similarly, when Darlene was an adolescent and constantly yelling at her mother, it was clear to her that it was not her fault. Her evaluation that the behavior was “not normal” is from her current perspective: “At that time I thought she was driving me crazy. ‘It’s not my fault.’ But when I look back on it, it was not normal.” Although Darlene used the term “driving me crazy,” it appears that she meant the term as it is used in common parlance, as “my mother is so irritating.”

One way to understand the normalization of the participants’ experiences prediagnosis is to examine how society generally avoided pathologizing the participants’ behaviors. For example, when Sarah attempted suicide as an adolescent, adults were angry with her, but they did not consider her crazy: “I wasn’t treated very well [at the hospital]. I think it was like a dumb teenager react—, reaction. They were mad at me. Everybody was mad at me.” Later, when Sarah was in college, she considered her mood fluctuations normal because “I bought into what the group of people around me thought [which] was that most of the way I was was because I studied all the time.”

Thus, the participants were aware of uncomfortable and painful feelings. We all experience mood fluctuations, anger, anxiety, and depression from time to time, so the participants did not view their experiences as more extreme than those of other people. As long as medical authorities (psychiatrists) and family members viewed the participants’ behaviors as normal, there was no reason to consider them otherwise. The external situating of self is consistent with Jacques Lacan’s ideas about the creation of the self. He postulates that it is only through the “gaze of the other” that one finds a label for self, even though that label always reflects the attitudes and desires of others (Lacan, 1966/1999). Lacan’s ideas on how others create the self, as articulated by Dor (1997), are as follows:

The child recognizes himself in his own [mirror] image only insofar as the other has already identified him with this image. He thus receives from the gaze of the other the confirmation that the image he perceives is indeed his. …. The ego … is irreducibly dependent on … the other (Dor, 1997, pp. 159–160).

Thus, participants’ evaluation of the normality of their feelings and behaviors depended on the assurances of doctors and family members.

### Early trauma

All the participants volunteered childhood history, which indicates that they make a connection between traumatic events in their childhoods and the bipolar diagnosis. All had been in psychotherapy, several with psychodynamically oriented therapists. Their perspectives might well have been influenced by the discourse in psychology around questions of attachment, attunement, and emotional regulation. Their childhood narratives involved trauma, emotional difficulties, problematic parental figures, and other challenges that, in both the medical model and psychodynamic conceptualizations, could impact a later diagnosis of bipolar disorder (Wyatt and Livson, 1994; Tarullo et al., 1995; Fonagy et al., 2002). For purposes of this analysis, the participants’ difficulties prediagnosis are significant in how they did *not* prepare the participants for receiving a diagnosis of bipolar disorder.

### Parental mental illness

Even though the participants did not view themselves as mentally ill, several described a parent that way. For example, Rose described her mother’s erratic moods and spending sprees at length. Rose said, “My mother was very, very depressed all the time. Um, if she wasn’t one way, she was another.” Rose called her mother “a thief and a liar and a cheat.” Yet Rose had only limited labels to make sense of her mother’s behavior, the primary one being that “mom was wacked.” Although her mother saw various psychiatrists, the only diagnosis was depression. Another explanation that people used in Rose’s world was menopause. Rose knew there was something more, but no one could explain it. Therefore, the labels available to explain Rose’s mother’s behavior were “wacked” (by the family), depression (by the authorities), or menopause (by the community).

Other participants also commented on parental behavior that they could not comprehend. As a young child, Natalie wondered why her mother literally drank herself to death a few years after the loss of a baby, and why she made no effort to stay alive for Natalie, who was then the youngest child. Kevin could never understand why his father clamped down on Kevin’s enthusiasm and then humiliated him for moments of eagerness. Darlene could not understand the behavior of her mother and grandmother who had “no boundaries.” Because the participants could not make sense of these parental behaviors that seemed erratic, unpredictable, and unexpected, they used the labels “wacked” or “crazy” to capture the sense that their parents’ behaviors were somehow outside the boundaries of normal.

### Initial negative reactions

When initially diagnosed, the participants’ first associations to the label of bipolar disorder were that they were crazy. The participants used colloquial and pejorative terms like “nuts,” “wacked,” “crazy,” “lithium-laced wack job,” and “yippy skippy,” rather than more generic terms, such as “ill,” “sick,” “troubled,” or “mental illness,” to describe their initial associations. They associated to the most psychotic and out of control manifestations of mania, referring to the extreme behaviors of people with bipolar disorder, such as “having sex with six guys or spending $20,000 … and jumping off roofs” (Darlene). These initial reactions seem to reflect societal associations to bipolar disorder as a significantly more serious classification than depression, a diagnosis with which most participants were familiar and comfortable.

These findings are noteworthy, especially considering that this is an articulate, well-educated, and socially sophisticated group. The participants had command of a rich and expressive vocabulary; their thinking was complex and nuanced; and their analyses were deep and insightful. Therefore, one might conclude that their use of pejorative colloquial language and their references to the most extreme manic behaviors are meaningful and interpretable.

Societal discourse continues to pathologize mental illness in general and bipolar disorder specifically, even in this era of public sensitivity about diversity. One example comes from a satire about political correctness. In response to the negative societal associations to words like “mad,” “madness,” “crazy,” “nuts,” and “wacked” (the very words participants used when they were first diagnosed), the National Alliance for the Mentally Ill (NAMI) and other organizations urged people to avoid use of such terms due to their negative connotations. In response, the satirist Borowitz (2007) mocked that position. His sardonic “news report” stated that the National Collegiate Athletic Association had changed the name of the March collegiate basketball tournament from “March Madness” to “March Bipolar Disorder,” inferring that the term “bipolar disorder” is synonymous with “madness.” The implication was that the only difference between the terms is that “madness” is an unacceptable, pejorative, unpolitically correct term whereas “bipolar disorder” is the proper term for the same construct.

Our society continues to be inundated with movies and cultural products that portray an exaggerated version of bipolar disorder and mental illness. Cultural images were even more charged in the second half of the twentieth century when the participants came of age, with movies such as *One flew over the cuckoo’s nest* and *Taxi driver*. Taking into account the power of these cultural images, it is understandable that when people are first labeled with bipolar disorder, they associate to psychosis, hospitalization, extremely low functioning, and exaggerated behaviors.

Further support for the societal association between bipolar disorder and mental illness is the fact that some of the participants had received the clear message from authorities (psychiatrists) that bipolar disorder is a much more serious disorder than depression. This occurred when participants were assured years earlier that they did not have bipolar disorder, as if it were a terrible fate narrowly avoided. Since the assurance was in the form of a negation, the impact of the affirmation (now, years later) makes the diagnosis feel even more serious. Darlene’s story is a representative one:

I remember that my doctor said, “Now I’m not putting you on lithium because you’re bipolar, because that’s not what I think.” Like—, he was like, “Don’t get worried, I’m not considering you bipolar. I just wanna try this lithium to help the Prozac.”

The unmistakable message was that bipolar disorder is of significant concern. With the new bipolar diagnosis, society and the medical establishment were now telling participants they have a significant disorder and are (or will become) crazy. The “gaze of the other” was now reflecting something much more serious.

With the diagnosis of bipolar disorder, all that changed was the public label. Participants had already been hospitalized for suicidal gestures or attempts, often years earlier. They had already experienced what came to be called mood fluctuations, anxiety, and depression; and they had already had episodes of sleeping excessively, crying uncontrollably, feeling extremely angry, or meeting endless people on the Internet, activities they later associated with bipolar disorder. For years, the participants had experienced the so-called symptoms that became the evidence and support for the bipolar label. Nonetheless, the impact of being assigned the bipolar label was life changing.

### A group relations analysis

The group relations perspective seems useful for conceptualizing why the impact of the bipolar label was so powerful. The label may have felt devastating if we view the participants as having fully incorporated or “swallowed whole” the negative societal associations linking bipolar disorder with madness. Their initial reactions were like a rote repetition of the stereotypes. Participants were all adults when diagnosed, so they had a lifetime to learn about crazy people who, until that moment, were “other,” and not self. Thus, the dilemma lies in incorporating what was formerly “not-me” into a sense of self. Psychodynamic group relations perspectives, including those of Volkan (1997), Sullivan (1953), Klein (1959/1985), and Bion (1959), provide a framework for understanding these processes.

In psychodynamic group relations understandings, the dominant group projects fearful and negative affects into a tainted outgroup. It then lodges in that group’s members those feared qualities (Volkan, 1997). Cultural rituals and objects reinforce the otherness and the inferiority of the outgroup’s members. The stereotyped negative qualities of outgroup members are absorbed by young children in an unchallenged manner. The process of incorporating undigested stereotypes is part of the developmental process when young children hear terms and ideas from their parents and treat them as absolute truth. Even older children will echo their parents’ off-hand comments, which they had incorporated in an unanalyzed manner. For example, my nephew Jacob, at age 11, announced that he would “have a midlife crisis” if he had to do homework over the weekend. He probably heard his mother use this phrase; he may not have fully understood its meaning, but he knew that it is associated with unpleasant angst. For the participants, however, what was undigested is much more serious than the notion of a midlife crisis.

Sullivan’s articulation of these processes focuses on the “not-me” experience. According to Sullivan, when an infant experiences unbearable anxiety in relationship to the “mothering one,” as Sullivan calls the parent, the child is unable to contain aspects of the experience. The terror of aggression, the shame of dependence, and the rage at imperfect parenting create strong feelings in the infant and, to alleviate suffering, these feelings become split-off and repressed, but still powerful, as “shadow,” or repressed knowledge. This affect becomes “not-me” and is out of conscious awareness and located in a nobody’s land that is the home of nightmares and terror movies. That which is unknown and uncontrollable inhabits that world – as well as crazy people who carry those dangerous qualities.

With regard to American notions of mental illness, we could say that the dominant group of “normal” people has created and maintained the group of “wacked” and “crazy” people as a denigrated other and has placed in that group all that is disquieting or frightening about mental illness (Bion, 1959). This might include the raging, unpredictable, psychotic, disorganized, and out of control “maniacs.” Bion explained that occasional contact with the others may confirm the stereotypes. We see homeless people who mutter to themselves or smell bad, for example, and we feel relieved that we are not in their shoes. Even if we occasionally have experiences that feel somewhat out of control, such as short-lived panic, depression, anger, or even suicidal ideation, we calm our anxiety by knowing that we are still not as deviant as those viewed as *really* crazy. With this understanding, we successfully avoid Emily Dickinson’s question, “Could it be Madness?— This?” (Dickinson, 1862/1960).

For the participants, being diagnosed with bipolar disorder represented moving from the dominant group of “normal” people into the group of “crazy maniacs.” Klein (1959/1985) postulated that one of the terrors humans face is that of being out of control. The bad or “wacked” parent in some of the participants’ narratives consisted of an unpredictable and out of control person. Following Klein further, it is possible that this represented a split in the participants themselves. Prior to the bipolar diagnosis, it was easier for participants to locate all of their own inconsistencies and momentary losses of control in their crazy, low functioning parents and not in themselves. The bipolar diagnoses forced them to confront these split-off aspects now as self.

The participants’ use of pejorative and childish language, like “wacked” “crazy,” and ‘yippy skippy” suggests that they had absorbed societal stereotypes about crazy people from an early age and had accepted these projections as truth. If we are unexpectedly labeled as one of the “others,” perhaps we first repeat to ourselves the terms that have always dictated what we know about *them*. If we find ourselves in the outgroup of mentally ill people, we now have to consider whether our assumptions about its members, such as being deranged, demented, unpredictable, and out of control, may also reside in us. We would likely experience shame and humiliation and feel defective from being associated in any way with that dangerous group.

Another way to understand the shock of the diagnosis is to consider that the participants did not previously have categories in which to place their experiences that psychiatrists considered symptoms of the manic or hypomanic aspects of bipolar disorder. Participants’ experiences of mood fluctuations, intense anger, or elevated moods had never before been pathologized; therefore, they were particularly vulnerable to the fear that they would now be cast unexpectedly into a socially denigrated group.

Coming to terms with the bipolar disorder label required that participants attempt to incorporate this new and troubling information into their self-understandings. As the participants digested the meaning of the diagnosis, they came to interpret their life experiences through the framework of bipolar disorder. They ultimately arrived at a kind of Kleinian depressive position in relation to the diagnosis, tolerating aspects of it while rejecting others.

Another way to conceptualize the denigration of people with mental illness is from Foucault’s (1954/1976, 1965/1988) perspective. Foucault theorized that society has placed people with mental illness, the “madmen,” in separated communities on the outside of the known world (such as on the Ship of Fools) or inside (in institutions). From that perspective, these findings suggest that, in present American society, people with mental illness continue to be separated from the dominant group but it is now accomplished through loading them up with denigrating labels and negative cultural metaphors rather than through physically expelling them.

The current effort in American society to honor diversity, including respect for people with mental illness, may fail to recognize that people labeled with mental illness may still be identified by society, albeit unconsciously, as a denigrated other. The participants’ strong reactions to the bipolar diagnosis suggests that unconscious societal forces that maintain people with mental illness outside the boundary of normal may be more powerful than society is willing to recognize or accept. As Volkan (1997) noted about ethnic conflict:

Humankind’s preoccupation with the other appears in ancient documents and in languages where the concept is elaborated with accrued connotations…. In the United States, the Apache Indians called themselves *indeh*, the people, and all others, *indah*, the enemy…. In English, the term *barbarian* refers to foreigners; in other words those who are uncivilized and ruthless and whose values differ from one’s own. … As W. H. Auden wrote … “if we did not have a hated ‘them’ to turn against, there would not be a loving ‘us’ to turn to” (25).

In other words, we need a category of out of control mentally ill people so that we can claim and even revel in our normality.

### Limitations

The proposed interpretations of the findings are consistent with the labeling, role, and stigma research that has found that negative and stigmatized attitudes toward mental illness are flourishing. The findings are also consistent with the research that people with mental illness continue to report stigmatizing and prejudicial experiences in employment and elsewhere. This analysis approached societal processes from a different angle, however. The goal was to make sense of participants’ narratives through psychodynamic theory and interpretive approaches that seek to understand *unconscious* aspects of participants’ verbal communications (Josselson, 2004).

The proposed interpretations about the meaning of participants’ language are hypotheses, not conclusions. Further, my questions or reactions as the interviewer may well have elicited certain kinds of responses. Moreover, the findings reflect the experiences of a small number of middle-class, educated Americans, mostly women, who identify as European American, and who were specifically chosen as participants because they had thought deeply about the meaning of the diagnosis. People from other backgrounds may share these or engage in other unconscious, societally inherited projections concerning people with bipolar disorder.

A final note is that the participants all came of age in the twentieth century. American culture may have changed sufficiently – and bipolar disorder may have become so much more commonplace now – that younger people might not react so negatively to a bipolar diagnosis. Recent research suggests that negative public attitudes toward depression may be abating (Angermeyer and Matschinger, 2003) and bipolar disorder may start following that trend. In light of all of these factors, the interpretations and conclusions are preliminary. The findings may be useful for thinking about how societal notions of mental illness may affect people who receive a bipolar diagnosis but they should not be used to generalize to a larger population.

## Conclusion

This paper contextualizes the initial shock and negative reactions of a group of people who had received a diagnosis of bipolar disorder in adulthood. Their initial reactions consisted of a terror that they now were or would become “crazy,” “nuts,” “mad,” or a “lithium-laced wack job.” These reactions are inconsistent with other aspects of the findings. One inconsistency is that the participants had all experienced noteworthy trauma and emotional difficulties throughout their lives prediagnosis. They had lived for years with other mental health diagnoses – five had been diagnosed with severe depression and one had been labeled an alcoholic. Several had been hospitalized with mental health symptoms, including three with suicidal gestures or attempts. A diagnosis of bipolar disorder, rather than depression, would have made sense as an explanation of participants’ overall experiences. Yet that was not the case initially for these participants.

The participants’ negative reactions, expressed in colloquial, almost childish, language, are also inconsistent with the participants’ education and professional backgrounds. Four out of six were mental health professionals – a doctor, two clinical psychologists, and a nurse-practitioner. All of the participants were extremely articulate and otherwise spoke in complex, sophisticated language.

I employed group relations approaches to develop interpretations of these initial reactions. I proposed that the participants had incorporated or “swallowed whole” the societal notions that bipolar disorder is a form of mental illness, in contrast to depression and alcoholism, their prior diagnoses. Like other members of American society, participants had apparently believed, unconsciously, that people with mental illnesses, including bipolar disorder, are out of control, deranged, “wacked,” and frightening. As members of the larger society, participants likely viewed people with mental illnesses as a denigrated other. To suddenly find themselves in this pathologized category must have been terrifying. Previously, many participants located all the “wackiness” in out of control parents. However, the new bipolar diagnosis required the participants to face the possibility that craziness, whatever that meant to them, might actually reside in them.

Participants’ belief that people with mental illnesses are a scapegoated other was buttressed by prior reactions of participants’ psychiatrists to their mental health conditions. Several participants had previously been reassured by their psychiatrists that their symptoms did *not* place them in the category of bipolar disorder (and by association, mental illness) but rather in a lesser, more “normal” category, namely, depression. Thus, when later diagnosed with bipolar disorder, the prior reassurances served to emphasize the seriousness of their present condition.

Over time, the participants had other reactions to the bipolar disorder label, even finding some relief at how it did organize and explain broad swaths of their and their parents’ prior experiences. Yet their initial reactions remain the focus of this examination.

The group relations perspectives speak to unconscious, unarticulated, and unacknowledged cultural processes that may be inconsistent with our society’s stated principles of inclusion, appreciation of diversity, and respect for people diagnosed with mental illness. Yet these underground communications remain critically important. As Rogers (2006) stated,

… in America we’ve watered down and neutralized Freud’s concept of the unconscious to such a degree that we no longer know how to listen as he listened. What’s taken its place is a practice that in fact closes down the unconscious and its great gifts to us. We diagnose, medicate, remove symptoms, change cognitions, change behavior, and understand relationships, and yet we ignore the unconscious—its otherness—because we’re frightened of it and have no access to it in the way we practice… (Rogers, 2006, p. 298).

I hope we can listen to the communications emerging from these participants’ reactions and then, like Dickinson 1862/1960), acknowledge and address our fears of our own madness.

## Conflict of Interest Statement

The author declares that the research was conducted in the absence of any commercial or financial relationships that could be construed as a potential conflict of interest.

## Footnotes

^1^This particular spelling of “wacked” is used because the term participants use seems closer to “wacky” than to the idiom “whacked out.” The term “wacky” is defined as meaning “odd or irrational; crazy” (Dictionary.com Unabridged, 2012).
